# DNA Polymorphisms and Biocontrol of *Bacillus* Antagonistic to Citrus Bacterial Canker with Indication of the Interference of Phyllosphere Biofilms

**DOI:** 10.1371/journal.pone.0042124

**Published:** 2012-07-27

**Authors:** Tzu-Pi Huang, Dean Der-Syh Tzeng, Amy C. L. Wong, Chun-Han Chen, Kuan-Min Lu, Ya-Huei Lee, Wen-Di Huang, Bing-Fang Hwang, Kuo-Ching Tzeng

**Affiliations:** 1 Department of Plant Pathology, National Chung-Hsing University, Taichung, Taiwan; 2 Department of Bacteriology, University of Wisconsin-Madison, Madison, Wisconsin, United States of America; 3 Department of Occupational Safety and Health, China Medical University, Taichung, Taiwan; Loyola University Medical Center, United States of America

## Abstract

Citrus bacterial canker caused by *Xanthomonas axonopodis* pv. *citri* is a devastating disease resulting in significant crop losses in various citrus cultivars worldwide. A biocontrol agent has not been recommended for this disease. To explore the potential of bacilli native to Taiwan to control this disease, *Bacillus* species with a broad spectrum of antagonistic activity against various phytopathogens were isolated from plant potting mixes, organic compost and the rhizosphere soil. Seven strains TKS1-1, OF3-16, SP4-17, HSP1, WG6-14, TLB7-7, and WP8-12 showing superior antagonistic activity were chosen for biopesticide development. The genetic identity based on 16S rDNA sequences indicated that all seven native strains were close relatives of the *B. subtilis* group and appeared to be discrete from the *B. cereus* group. DNA polymorphisms in strains WG6-14, SP4-17, TKS1-1, and WP8-12, as revealed by repetitive sequence-based PCR with the BOXA1R primers were similar to each other, but different from those of the respective *Bacillus* type strains. However, molecular typing of the strains using either tDNA-intergenic spacer regions or 16S–23S intergenic transcribed spacer regions was unable to differentiate the strains at the species level. Strains TKS1-1 and WG6-14 attenuated symptom development of citrus bacterial canker, which was found to be correlated with a reduction in colonization and biofilm formation by *X. axonopodis* pv. *citri* on leaf surfaces. The application of a *Bacillus* strain TKS1-1 endospore formulation to the leaf surfaces of citrus reduced the incidence of citrus bacterial canker and could prevent development of the disease.

## Introduction

*Bacillus* species are natural inhabitants of the phyllosphere [1] and rhizosphere [2]. They form endospores and various strains are capable of producing enzymes, antibiotics, proteins, vitamins or secondary metabolites that exhibit the ability to promote growth or induce defense mechanisms in animals and plants [3]. Thus, *Bacillus* species are important candidates for microbial control agents for plant diseases and pests [2], [4], [5], [6], protectants for seeds [7], and probiotics [8]. *Bacillus* species have been shown to suppress plant diseases caused by diverse microorganisms including *Phytophthora medicaginis* [9], *Pythium torulosum* [10], *Botrytis cinerea* [11], *Rhizoctonia solani* [12], *Sclerotinia sclerotiorum* [12], *Colletotrichum gloeosporioides* [2], *Colletotrichum orbiculare* [13], *Fusarium* spp. [12], [14], *Phytophthora sojae*, *Cronartium quercuum* f. sp. *fusiforme* [15], *Xanthomonas oryzae* [16], [17], *Pseudomonas syringae* [13], [18], and *Ralstonia solanacearum* [19]. Moreover, known *Bacillus* species have been used for the development of biocontrol agents including, but are not limited to, *B. subtilis* [2], [11], [14], [17], [18], [19], *B. amyloliquefaciens* [12], [20], *B. cereus* [9], [10], *B. megaterium* [6], *B. pumilus* [13], [15], [17], and *B. thuringiensis* [5]. However, a few *Bacillus* species are known to produce enterotoxins that may cause human illness [21]. The development of promising biocontrol products, such as several *Burkholderia cepacia* complex strains that have been registered by the United States Environmental Protection Agency for use as microbial pesticides has been terminated because of concerns over infections among immunocompromised humans [22]. Thus, identification and selection of ‘generally recognized as safe’ (GRAS) organisms prior to the intensive development process required for biocontrol agents is recommended.

*Bacillus* species are genotypically diverse organisms. The comparison of small-subunit ribosomal RNA sequences reveals the presence of five genetically distinct groups in the genus [23]. Those *Bacillus* strains that are known to have the potential to protect plants from pathogens or pests or stimulate plant growth are attributed to two groups, the *B. cereus* group and the *B. subtilis* group. The *B. cereus* group includes *B. anthracis*, *B. cereus*, *B. thuringiensis*, *B. mycoides*, *B. pseudomycoides*, and *B. weihenstephanesis*; the *B. subtilis* group includes *B. subtilis*, *B. pumilus*, *B. atrophaeus*, *B. licheniformis* and *B. amyloliquefaciens* [23]. Many *Bacillus* species are generally considered harmless, and *B. subtilis* has even been granted GRAS status by the United States Food and Drug Administration (US FDA). However, *B. anthracis* can cause anthrax in humans and cattle, and *B. cereus* is known to produce enterotoxins that cause food poisoning [21]. Molecular techniques, including 16S rRNA gene sequencing, DNA polymorphism analyses by tDNA-PCR for the tDNA-intergenic spacer region, ITS-PCR for the 16S–23S intergenic transcribed spacer region, and repetitive element sequence-based PCR (rep-PCR) using the ERIC2, BOXA1R and (GTG)_5_ primers [24], [25], [26], have been developed for rapid species identification of the *Bacillus* genus.

Citrus fruits are of economic importance worldwide [27], [28]. The major bacterial disease of citrus, citrus bacterial canker, is caused by *X. axonopodis* pv. *citri* [29], for which the currently published nomenclature is *X. citri* subsp. *citri* [30]. To control this disease, copper salts and antibiotics are suggested [31]; however, several *Xanthomonas* strains have been found to both of these methods [32]. Thus, the development of alternative control strategies for this disease is necessary.

Microbial communities attached to a surface are referred to as biofilms [33]. The synergistic or antagonistic interactions between biofilm organisms and their respective hosts can contribute to the successful establishment of symbiotic or pathogenic relationships [34]. Consequently, interfering with bacterial biofilm formation has been suggested as a novel strategy for disease control [35], [36], [37]. It has been shown that biofilm formation was necessary for epiphytic fitness and canker development by the phytopathogen *X. axonopodis* pv. *citri* [38]. For the beneficial antagonist, root colonization plays a key role in the interaction of *B. subtilis* with Arabidopsis and the pathogen *Pseudomonas syringae* [18]. Our previous study indicated that antagonistic *B. amyloliquefaciens* WG6-14 was a potential biopesticide for controlling citrus bacterial canker (unpublished data), and an endospore formulation of this antagonist has been officially recommended for controlling bakanae disease of rice in Taiwan. However, the interaction of *X. axonopodis* pv. *citri* and antagonistic *Bacillus* species in the phyllosphere of citrus has not been investigated.

In this study, native bacilli isolated from potting mixes, organic compost, and soil in Taiwan were assessed for antagonistic activity against citrus canker bacteria. The genetic identities determined by rDNA sequences of bacilli from Taiwan, their respective type strains, and other industrial strains were compared. DNA polymorphisms were determined by molecular typing of the 16S–23S intergenic transcribed spacer region, tDNA intergenic spacer length analysis and repetitive element sequence-based PCR. In addition, the efficacy of reducing disease incidence by application of *Bacillus* species and the interaction between the antagonist and the pathogen in the phyllosphere of citrus were investigated.

## Results

### *Bacillus* strains exhibited antagonistic activity against the pathogen of citrus bacterial canker

**Figure 1:**
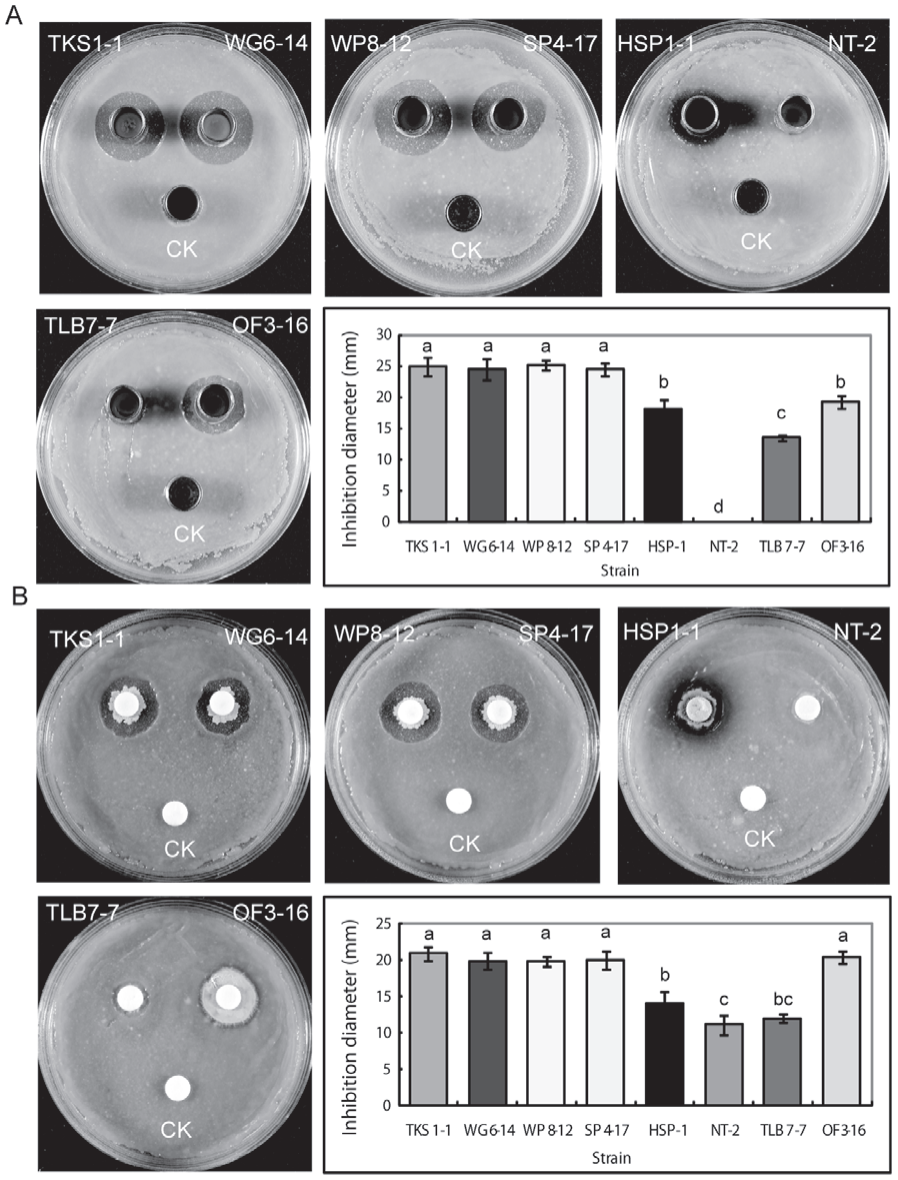
Antagonistic activity of *Bacillus* species against *X. axonopodis* pv. *citri* XW19. A 20 µl aliquot of *X. axonopodis* pv. *citri* XW19 suspension (OD_620_ = 0.3) was spread on an SYB agar plate. Following overnight incubation at 30°C, 20 µl of *Bacillus* suspension (OD_620_ = 0.3) was spotted (A) inside the stainless steel ring or (B) on a paper disc. The plates were incubated at 30°C, for 5 days. CK, 20 µl of sterile Milli-Q water was used as a control. The results represent the means and standard deviations (error bars) of a representative experiment. Different lowercase letters indicate significant differences (p<0.05) according to Tukey's HSD test.

*Bacillus* strains with a broad spectrum of antagonistic activity against various phytopathogens including *Pythium aphanidermatum*, *Rhizoctonia solani* AG4, *Xanthomonas axonopodis* pv. *vesicatoria* XVT12 and *X. axonopodis* pv. *citri* XW19 were isolated from plant potting mixes, organic compost and soil samples collected from the field (data not shown). Seven of the 326 strains tested (HSP1, TKS1-1, OF3-16, SP4-17, WG6-14, TLB7-7, and WP8-12) that showed superior antagonistic activity, along with one other strain (NT-2 isolated from natto, a Japanese fermented soybean product), were used in this study. According to a dual culture assay using stainless steel rings, strains TKS1-1, WG6-14, WP8-12 and SP4-17 exhibited significantly higher antagonistic activity against *X. axonopodis* pv. *citri* XW19 than strains HSP-1, NT-2, TLB7-7, and OF3-16 (Fig. 1 A). The antagonistic activity of strain OF3-16 on paper discs was similar to that of strains TKS1-1, WG6-14, WP8-12, and SP4-17 (Fig. 1 B).

### Sequence and phylogenetic analyses of 16S rRNA genes in native *Bacillus* species

To identify the *Bacillus* strains, each strain was subjected to physiological and biochemical characterization using the methods described in Bergey's Manual of Systematic Bacteriology [39] and was identified using the Biolog system (Biolog Inc., CA, USA). The physiological and biochemical tests included Gram staining, endospore staining, starch hydrolysis, Voges-Proskauer test, the oxidase-fermentation test, gelatin hydrolysis, citrate utilization, nitrate reduction, arginine dihydrolase activity, growth in 7% sodium chloride and growth at 50°C. Strains HPS-1, OF3-16, SP4-17, TKS1-1, WP8-12, and WG6-14 all showed positive reactions for these tests were classified as *B. licheniformis* (data not shown). Strain TLB7-7 did not hydrolyze starch or reduce nitrate and was classified as *B. pumilus* (data not shown). The results of the Biolog analysis indicated that strain HSP-1 was *B. licheniformis*, strain SP4-17 was *B. megaterium*, strains TKS1-1 and WP8-12 were *B. subtilis*, strain TLB7-7 was *B. pumilus* and strain WG6-14 was *B. amyloliquefaciens*; strain OF3-16 could not be classified using the Biolog system (Table 1). Thus, the species attributes for most of the strains were designated based on Biolog analysis except for strain OF3-16, which was based on the physiological and biochemical characteristics described in Bergey's Manual.

**Table 1:** Bacterial strains and plasmids used in this study.

Strains/Plasmids	Relevant characteristics^a^	Source or reference^b^
***Bacillus***		
ATCC 23842	*B. amyloliquefaciens* type strain, 16S rRNA sequence accession no. EU689157	NCBI
BCRC 11601	*B. amyloliquefaciens* type strain ATCC 23350, DSM7	BCRC
WG6-14	*B. amyloliquefaciens* from rhizosphere soil, Wufong, Taiwan	This study
ATCC 14579	*B. cereus* type strain, 16S rRNA sequence accession no. AF290547	NCBI
ATCC 11778	*B. cereus*, 16S rRNA sequence accession no. AF290546	NCBI
UW85	*B. cereus* isolated from alfalfa plant root	[9]
BCRC 11702	*B. licheniformis* type strain ATCC14580, 16S rRNA sequence accession no. NC_006270	BCRC, NCBI
HSP-1	*B. licheniformis* from plant potting mix, Puli, Taiwan	This study
OF3-16	*B. licheniformis* from organic compost, Changhwa, Taiwan	This study
BCRC 10608	*B. megaterium* type strain DSM 32, 16S rRNA sequence accession no. X60629	BCRC, NCBI
DSM 319	*B. megaterium*, 16S rRNA sequence accession no. NC_014103	NCBI
SP4-17	*B. megaterium* from rhizosphere soil, Taichung, Taiwan	This study
ATCC 6462	*B. mycoides*, 16S rRNA sequence accession no. EF210295	NCBI
BCRC 11706	*B. pumilus* type strain ATCC 7061, 16S rRNA sequence accession no. AY876289	BCRC, NCBI
TLB7-7	*B. pumilus* from rhizosphere soil, Tali, Taiwan	This study
BCRC 10255	*B. subtilis* subsp. *subtilis* type strain ATCC6051, DSM 10, 16S rRNA sequence accession no. AJ276351	BCRC, NCBI
BCRC 80045	*B. subtilis* subsp. *spizizenii* type strain ATCC 6633, 16S rRNA sequence accession no. AB018486	BCRC, NCBI
TKS1-1	*B. subtilis* from plant potting mix, Puli, Taiwan	This study
NTA-1	*B. subtilis* from natto	This study
NT-2	*B. subtilis* from natto	This study
NTB-1	*B. subtilis* from natto	This study
WP8-12	*B. subtilis* from rhizosphere soil, Wuri, Taiwan	This study
BT407Cry^−^	*B. thuringiensis* lacking crystalline endotoxin	[54]
***Xanthomonas axonopodis*** ** pv. ** ***citri***		
XW19	Wild type	[55]
TPH2	Gm^r^, XW19 harboring pBBR1MCS5	This study
**Plasmid**		
pBBR1MCS5	Gm^r^, broad host range cloning vector	[56]
pGTKan	Gm^r^, pPROBE-GTkan containing a 131-base pair *npt*II promoter fragment from Tn5 and fused to *gfp*	[57]

a Species attributes of native *Bacillus* strains were identified based on Biolog analysis except for strain OF3-16, which was identified based on physiological and biochemical characteristics described in the Bergey's Manual of Systematic Bacteriology [39].

b ATCC, American Type Culture Collection Center; BCRC, Bioresource Collection and Research Center, Taiwan; DSM, Leibniz Institute DSMZ-German Collection of Microorganisms and Cell Cultures; NCBI, National Center for Biotechnology Information.

**Figure 2:**
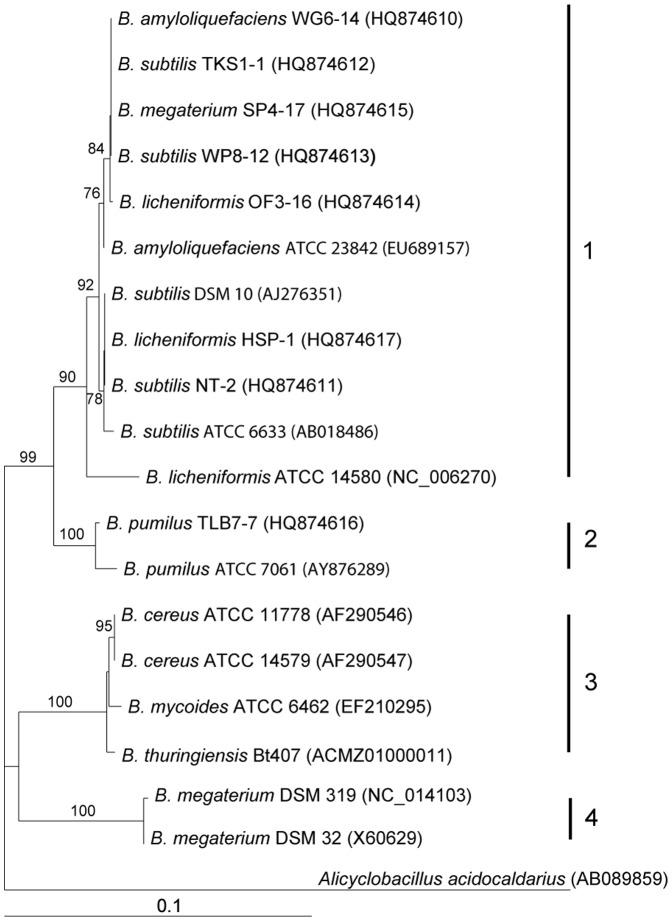
Phylogenetic tree of *Bacillus* species based on 16S rRNA gene sequences. The tree was constructed using the neighbor-joining method and genetic distances were generated using the Kimura 2-parameter method. The numbers at the branches are bootstrap confidence percentages from 1000 bootstrapped trees. *Alicyclobacillus acidocaldarius* (GenBank accession no. AB089859) was used as the outgroup. The numbers in parentheses indicate the GenBank accession numbers.

For phylogenetic analysis, partial 16S rRNA gene sequences were PCR amplified from eight native *Bacillus* strains: WG6-14, TKS1-1, SP4-17, WP8-12, OF3-16, HSP-1, NT-2, and TLB7-7. Except for strain TLB7-7, which was classified in the same clade as the *B. pumilus* type strain (ATCC 7061), the remaining seven strains formed a cluster with the type strains of *B. amyloliquefaciens* (ATCC 23842), *B. subtilis* (DSM 10), *B. subtilis* (ATCC 6633) and *B. licheniformis* (ATCC 14580) (Fig. 2). The sequence identity of the 16S rRNA sequences from strains WG6-14, TKS1-1, SP4-17, WP8-12, and OF3-16 was 99%; that from HSP-1 and NT-2 was 100%; and that from TLB7-7 was 97% with *B. subtilis* DSM 10 (data not shown); and that from TLB7-7 was 99% with *B. pumilus* type strain ATCC 7061 (data not shown). These results suggest that the isolated *Bacillus* strains native to Taiwan that showed substantial antagonistic activity against *X. axonopodis* pv. *citri* are close relatives of the *B. subtilis* group including *B. subtilis, B. pumilus*, *B. licheniformis* and *B. amyloliquefaciens*, and that they are distant from strains of the *B. cereus* group including *B. cereus*, *B. mycoides* and *B. thuringiensis*.

### ITS-PCR, tDNA-PCR, and rep-PCR fingerprint and cluster analysis of *Bacillus* species

**Figure 3:**
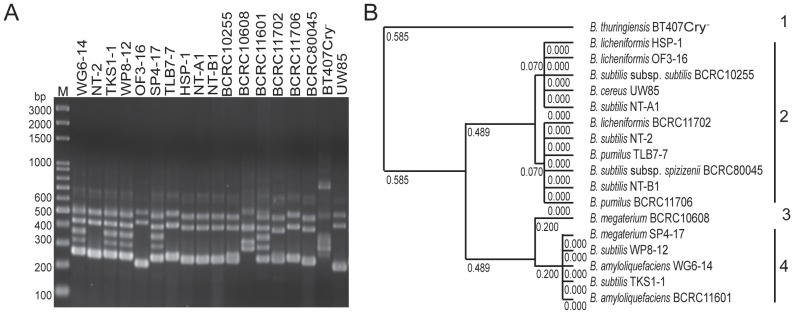
ITS-PCR fingerprint and UPGMA cluster analysis of *Bacillus* species. (A) ITS-PCR fingerprint and (B) UPGMA cluster analysis. The UPGMA cluster analysis was based on ITS-PCR. M, GeneRuler™ 100 bp plus DNA ladder (Fermentas, Taipei, Taiwan).

ITS-PCR, tDNA-PCR and rep-PCR fingerprinting have been used to differentiate isolates among a wide range of bacterial and fungal genera and species as well as to study genomic diversity [24], [40], [41], [42]. To evaluate the DNA polymorphisms of *Bacillus* species native to Taiwan and their respective type stains, ITS-PCR using the primers L1 and G1 to amplify the 16S–23S intergenic transcribed spacer region, tDNA-PCR using the primers T5A and T3B to amplify the tDNA-intergenic spacer region, and rep-PCR analyses using the primers ERIC2, BOXA1R and (GTG)_5_ as described by Freitas *et al.* [24] were performed. DNA polymorphisms were assessed four times with reproducible results. ITS-PCR fingerprinting and unweighted pair group method with arithmetic mean (UPGMA) cluster analysis classified all tested strains into 4 distinct groups. *Bacillus* strains SP4-17, WP8-12, WG6-14, and TKS1-1, which showed the greatest antagonistic activity, were in a cluster with the *B. amyloliquefaciens* type strain BCRC11601 (Fig. 3). However, the reference strains *B. subtilis* subsp. *subtilis* BCRC10255, *B. licheniformis* BCRC11702, *B. subtilis* subsp. *spizizenii* BCRC80045, *B. pumilus* BCRC11706, and *B. cereus* UW85; the strains NT-A1, NT-B1 and NT-2 isolated from Japanese natto and the native strains HSP1, OF3-16, and TLB7-7 all showed the same ITS-PCR fingerprint patterns. These results suggest that ITS-PCR fingerprint analysis was not able to differentiate *Bacillus* isolates at the species level and discriminate *B. subtilis* from *B. cereus*.

**Figure 4:**
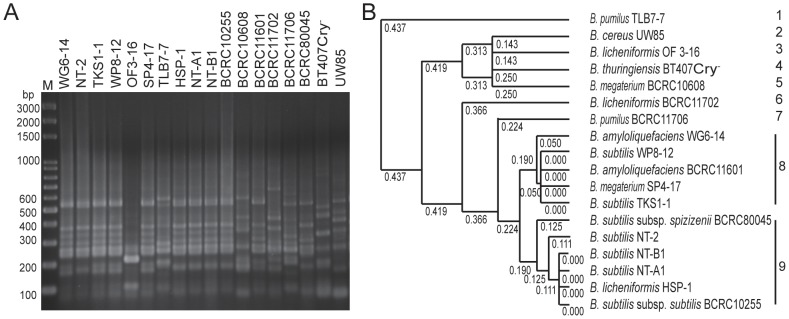
tDNA-PCR fingerprint and UPGMA cluster analysis of *Bacillus* species. (A) tDNA-PCR fingerprint and (B) UPGMA cluster analysis. The UPGMA cluster analysis was based on tDNA-PCR. M, GeneRuler™ 100 bp plus DNA ladder (Fermentas, Taipei, Taiwan).

Using tDNA-PCR fingerprinting, the tested strains showed nine pattern types (Fig. 4). Strains WG6-14, WP8-12, SP4-17 and TKS1-1 were homologous and showed the same DNA banding pattern as *B. amyloliquefaciens* BCRC11601; strains NT-2, NT-B1, NT-A1 and HSP-1 were homologous and showed the same DNA banding pattern as the *B. subtilis* type strains BCRC80045 and BCRC10255. Strains TLB7-7 and OF3-16 were designated as *B. pumilus* and *B. licheniformis*, respectively, according to Biolog analysis, 16S rRNA sequence analysis and physiological and biochemical characterization. These strains showed tDNA-PCR fingerprints that were distinct from their respective type strains.

**Figure 5:**
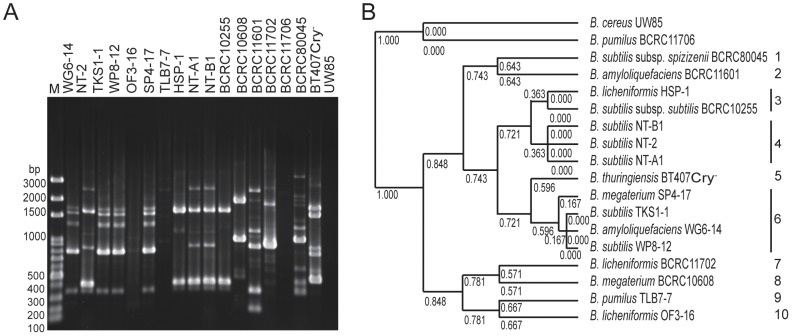
BOXA1R-PCR fingerprint and UPGMA cluster analysis of *Bacillus* species. (A) BOXA1R-PCR fingerprint and (B) UPGMA cluster analysis. The UPGMA cluster analysis was based on BOXA1R-PCR using the BOXA1R primer. M, GeneRuler™ 100 bp plus DNA ladder (Fermentas, Taipei, Taiwan).

Three sets of primers, ERIC2, (GTG)_5_ and BOXA1R [40], were used for rep-PCR fingerprint analysis. Based on BOXA1R-PCR fingerprint analysis, ten banding patterns were observed (Fig. 5). Strain HSP-1 showed the same pattern as *B. subtilis* subsp. *subtilis* BCRC10255. Strains NT-B1, NT-2 and NT-A1 isolated from natto formed a cluster that was different from that of their close relatives, strains *B. subtilis* subsp. *subtilis* BCRC10255 and *B. subtilis* subsp. *spizizenii* BCRC80045. Strains SP4-17, TKS1-1, WG6-14 and WP8-12 were homologous and showed a unique banding pattern. Negative results were observed with BOXA1R-PCR fingerprinting for strains *B. cereus* UW85 and *B. pumilus* BCRC11706. Both ERIC2-PCR and (GTG)_5_-PCR amplification were negative for most of the tested strains (data not shown). Of the three primer sets, BOXA1R-PCR showed unique patterns that could differentiate strains native to Taiwan from the reference strains. Moreover, strains SP4-17, TKS1-1, WG6-14 and WP8-12, which showed superior antagonistic activity against *X. axonopodis* pv. *citri* (Fig. 1), had the same BOXA1R-PCR fingerprint, which was distinct from those of all reference strains.

### Attenuated symptom development of citrus bacterial canker by treatment with *B. subtilis* and *B. amyloliquefaciens*

**Figure 6:**
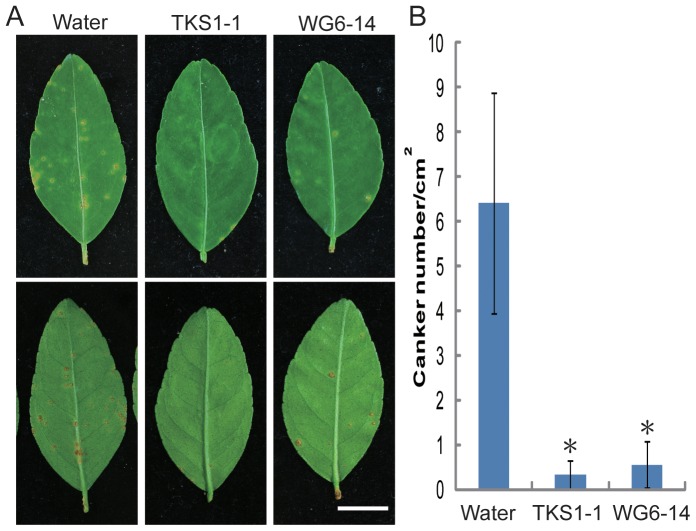
The effect of *B. subtilis* TKS1-1 and *B. amyloliquefaciens* WG6-14 on the disease severity of citrus bacterial canker on Mexican lime. (A) Symptoms on upper (top panels) and lower (bottom panels) leaf surfaces of Mexican lime one month post -inoculation with *X. axonopodis* pv. *citri* TPH2 (OD_620_ = 0.3). Milli-Q water or *B. subtilis* TKS1-1 (TKS1-1) and *B. amyloliquefaciens* WG6-14 (WG6-14) culture suspensions were sprayed on the leaves of Mexican lime one day prior to inoculation with *X. axonopodis* pv. *citri* TPH2. (B) Number of cankers per cm^2^ on each leaf. All experiments were performed three times with similar results. The results are the means and standard deviations (error bars) of five replicates from one representative experiment. *, significantly different (p<0.05) from water control analyzed by one-way ANOVA and Tukey's HSD test. Scale bar, 1 cm.

Our previous results indicated that the application of *B. amyloliquefaciens* WG6-14 endospores one day prior to inoculation with citrus canker bacteria reduced disease incidence from 97.7% to 3.03% (unpublished data). To assess the effect of *B. subtilis* TKS1-1 and *B. amyloliquefaciens* WG6-14 on the disease severity of citrus bacterial canker, *Bacillus* suspensions (overnight cultures diluted to an OD_620_ of 0.3, ca. 10^8^ CFU/ml) were sprayed on the leaves of Mexican lime 1 day prior to inoculation with *X. axonopodis* pv. *citri* TPH2 (overnight cultures diluted to an OD_620_ of 0.3, ca. 10^8^ CFU/ml), and the number of cankers per cm^2^ on each leaf with and without *Bacillus* treatment was determined. Less severe canker symptoms or no symptom were observed on the *Bacillus*-treated leaves compared to the water control (Fig. 6 A). The number of cankers per cm^2^ for the untreated control was 6.4±2.5, compared to 0.3±0.3 and 0.6±0.5 for the *B. subtilis* TKS1-1 and *B. amyloliquefaciens* WG6-14 treatments, respectively (Fig. 6 B). The number of cankers per cm^2^ developing following with the application of *Bacillus* suspensions was significantly reduced by up to 6-fold (p<0.05).

### The effect of *Bacillus* on colonization and biofilm formation by citrus canker bacteria on leaf surfaces

**Figure 7:**
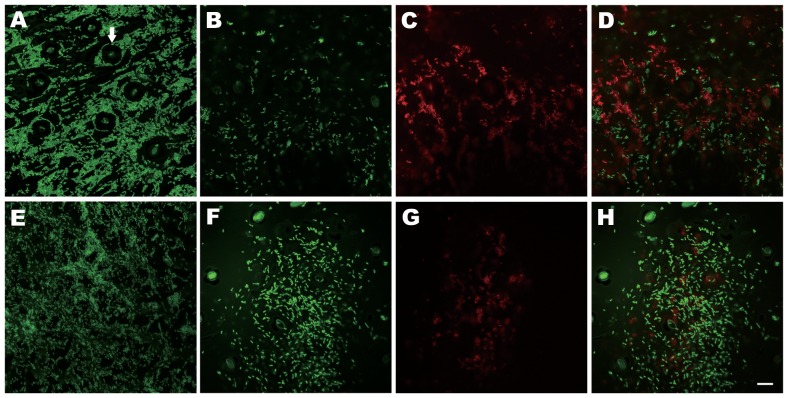
Colonization by *X. axonopodis* pv. *citri* strain TPH2 and *B. subtilis* TKS1-1 and *B. amyloliquefaciens* WG6-14 on leaf surfaces of Mexican lime observed by confocal laser scanning microscopy. Leaf surfaces were spray inoculated with (A, E) *X. axonopodis* pv. *citri* strain TPH2 harboring pGTKan, and (B, C) *B. subtilis* strain TKS1-1, or (F, G) *B. amyloliquefaciens* strain WG6-14 1 day prior to inoculation with *X. axonopodis* pv. *citri* strain TPH2 harboring pGTKan. The photos were taken 1 day post-inoculation of the pathogen. Green, *X. axonopodis* pv. *citri* strain TPH2 expressing green fluorescent protein. Red, acridine orange stained cells. (D) and (H), merged images of (B, C) and (F, G), respectively. Arrow, stomata. Scale bar, 10 µm.

According to Rigano *et al.* [38] and our previous findings (unpublished data), biofilm formation is important for epiphytic survival and the development of canker disease. Colonization of the leaf surfaces of Mexican lime by *X. axonopodis* pv. *citri* strain TPH2 harboring a green fluorescent protein expressing plasmid, pGTKan (Table 1), was examined by confocal laser scanning microscopy. Individual cells attached to the surfaces of leaves submerged in bacterial suspension (overnight cultures diluted to an OD_620_ of 0.05 in trypticase soy broth) were observed 1 day post-inoculation, and microcolony and biofilm development were observed after 2 days (data not shown). Biofilms consisting of multicellular aggregates similar to those observed by Rigano et al. [38] were observed 1 day post-inoculation with *X. axonopodis* pv. *citri* strain TPH2 harboring pGTKan on the leaf surfaces of Mexican lime grown in the greenhouse (Fig. 7 A and E). Bacterial aggregates could be observed surrounding and inside the stomata (Fig. 7 A). Treatment with *B. subtilis* strain TKS1-1 or *B. amyloliquefaciens* strain WG6-14 resulted in fewer *X. axonopodis* pv. *citri* cells attaching to the leaf surface compared to no treatment, and the cells were dispersed (Fig. 7 B and F, respectively). *B. subtilis* strain TKS1-1 and *B. amyloliquefaciens* strain WG6-14 cells were stained with acridine orange and showed red fluorescence (Fig. 7 C and G, respectively). The combined green and red fluorescent images indicated that small aggregates of *Bacillus* cells (red) were scattered around the *X. axonopodis* pv. *citri* cells (green) (Fig. 7 D and H). These results suggest that by spraying antagonistic *Bacillus* 1 day prior to inoculation with the pathogen, colonization and biofilm formation by citrus canker bacteria on leaf surfaces could be reduced.

### *Bacillus* endospore formulations are effective in reducing the development of canker symptoms and the incidence of citrus bacterial canker disease

**Figure 8:**
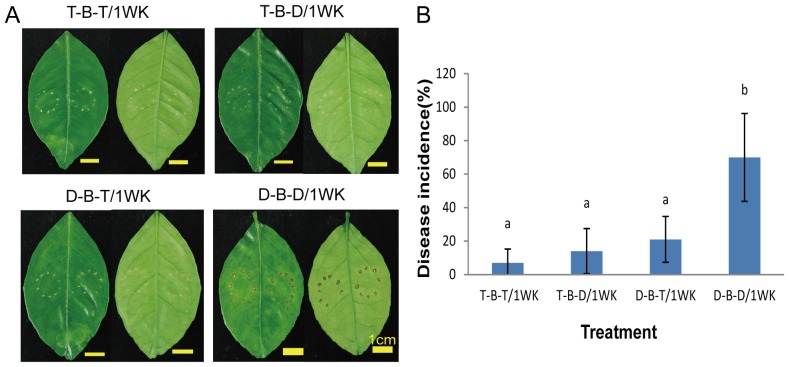
The effect of application time and frequency of *B. subtilis* strain TKS1-1 on symptom development and disease incidence of citrus bacterial canker on navel orange grown in the greenhouse. *B. subtilis* strain TKS1-1 endospore formulation (T, 10^9^ CFU/ml) and *X. axonopodis* pv. *citri* XW19 (B, 10^8^ CFU/ml) were used. Treatment T-B-T/1WK, strain TKS1-1 endospores were applied 24 h prior to inoculation with *X. axonopodis* pv. *citri* XW19, and then weekly post-pathogen inoculation for 4 weeks; treatment T-B-D/1WK, strain TKS1-1 endospores were applied 24 h prior to *X. axonopodis* pv. *citri* XW19 inoculation; treatment D-B-T/1WK, strain XW19 was inoculated, then strain TKS1-1 endospores were sprayed every week post-pathogen inoculation for 4 weeks; treatment D-B-D/1WK, only strain XW19 was inoculated. Without *Bacillus* treatment, Milli-Q water (D) was sprayed on the leaf surface. (A) Symptoms on upper (left) and lower (right) leaf surfaces after different treatments. Scale bars, 1 cm. (B) Disease incidence was rated 4 weeks post-inoculation. Bars indicate standard deviations. Columns that are top-labeled with different letters are significantly different (p<0.05) according to one-way ANOVA and Tukey's HSD test.

*B. subtilis* TKS1-1 endospore formulations were applied to the leaves of navel orange trees grown in the greenhouse to assess disease control efficacy for citrus bacterial canker. The results indicated that the spray-application of an endospore formulation diluted 100-fold (final concentration ca. 10^9^ spores/ml) was effective in reducing symptom development and disease incidence of citrus bacterial canker compared to no treatment (Fig. 8). The efficacy of treatments applied 24 h prior to pathogen inoculation and treatments applied post-inoculation on reducing disease incidence was similar, and was not significantly affected by the frequency of application.

## Discussion

No known biocontrol agents have been developed for the disease management of citrus bacterial canker. To explore the potential of bacilli native to Taiwan to control this disease, *Bacillus* species with a broad spectrum of antagonistic activity against various phytopathogens were isolated from potting mixes, organic compost and rhizosphere soils. By dual culture assay, seven strains TKS1-1, OF3-16, SP4-17, HSP1, WG6-14, TLB7-7, and WP8-12 showing superior antagonistic activity were chosen for biopesticide development and for further investigation. Using established and patented methods, we mass-produced strain TKS1-1 endospores, and showed them to be effective in reducing the severity and incidence of citrus bacterial canker. In addition, an endospore formulation of strain WG6-14 reduced bacterial black spot of mango and bacterial leaf blight of rice (unpublished data). Endospore formulations of *Bacillus* strain WG6-14 have been commercialized and registered as biocontrol agents for rice bakanae disease in Taiwan. As part of the safety requirements for biopesticide development, GRAS organisms are preferred as biocontrol agents. Our results, based on physiological and biochemical characteristics, 16S rDNA sequences and tDNA-PCR analyses, indicate that all seven native strains with antagonistic activity against *X. axonopodis* pv. *citri* and that demonstrated high efficacy in suppressing citrus bacterial canker disease were in the same clades as the type strains of the *B. subtilis* group that are listed as GRAS bacteria by the US FDA and that are distinct from strains of the *B. cereus* group [23].

ITS-PCR, tDNA-PCR and rep-PCR analyses have been successfully used to investigate the species and intraspecific variability of *Bacillus* species [24], [26], [41], [43]. Of these molecular typing techniques, all of which were used in this study, rep-PCR analysis using the BOXA1R primer displayed the best resolving power for discriminating between native strains exhibiting superior antagonistic activity against *X. axonopodis* pv. *citri* and the reference strains. ITS-PCR analysis was not sufficient to distinguish strains of the *B. subtilis* group from *B. cereus* strain UW85 [23]. This result suggests that ITS-PCR analysis was not adequate for discriminating between *Bacillus* strains at the species level as was demonstrated by Freitas *et al.* [24]. In contrast, Wunschel *et al.* showed that the banding patterns generated by PCR analysis of the rRNA spacer region could distinguish *B. subtilis* from species in the *B. cereus* group but could not differentiate between species within the *B. cereus* group [25]. On the basis of cell wall constituents and DNA-DNA relatedness data, *B. subtilis* strains were reclassified into two subspecies: *B. subtilis* subsp. *subtilis* and *B. subtilis* subsp. *spizizenii* [44]. Our data indicate that these two subspecies were grouped into one cluster by tDNA-PCR analysis and two clusters by BOXA1R-PCR analysis. In addition, DNA polymorphisms in strains WG6-14, SP4-17, TKS1-1, and WP8-12, as revealed by rep-PCR using the BOXA1R primer, were similar to each other, but different from their respective type strains. These four strains were associated with the greatest antagonistic activity. Our results suggest that the DNA fingerprint generated with BOXA1R-PCR could be valuable not only for patenting or commercializing these *Bacillus* strains, but also for creating markers for the selection of antagonists against *X. axonopodis* pv. *citri*.

Epiphytic and root colonization are considered as the process of biofilm formation [45]. Bacterial biofilm formation has been shown to be necessary for epiphytic fitness, pathogenesis, antagonism and symbiosis with the host organism [34]. Thus, microbial infection control strategies could be developed based on interfering with biofilm formation [35], [36], [37]. Rigano *et al.* [38], as well as our unpublished data, demonstrated that biofilm formation by *X. axonopodis* pv. *citri* on the leaf surfaces of citrus was associated with the occurrence of citrus canker symptoms. Here, we investigated the efficacy of using antagonistic bacilli to interfere with this process. Our data indicate that biofilm development by *X. axonopodis* pv. *citri* on the leaf surfaces of Mexican lime was reduced following spray inoculation of *Bacillus* strains WG6-14 and TKS-1 1 day prior to pathogen inoculation when compared to no treatment. Application of these two *Bacillus* strains to citrus leaves resulted in reduced symptom development, suggesting that these antagonistic bacilli are potential biocontrol agents for citrus bacterial canker disease. Taken together, these results suggested that control may be associated with the interference of colonization and biofilm formation by *X. axonopodis* pv. *citri* in the phyllosphere, which is the site of initial colonization and infection. The biocontrol efficacy of citrus canker disease by *Bacillus* strain TKS1-1 was further demonstrated by spray application of endospore formulations in the greenhouse. Rhizosphere-colonizing *B. subtilis* 6051 forms a stable biofilm and secretes surfactin, which work together to protect Arabidopsis plants from infection by pathogenic *P. syringae* [18]. We did not exclude the possibility that *Bacillus* strains TKS1-1 and WG6-14 also may secrete surfactin [18], bacteriocins such as xanthobacidin [46], or other cyclic lipopeptides [47] because both strains inhibited the growth of *X. axonopodis* pv. *ciri* XW19. In addition, cyclic lipopeptides are reportedly involved in biofilm formation by *Bacillus* species [18], [45], [48]. Alternatively, some *Bacillus* species including *B. subtilis*, *B. amyloliquefaciens*, *B. pumilus*, *B. mycoides*, *B. pasteurii*, *B. thuringiensis*, and *B. cereus* apparently induce plant resistance [44]. Determinants for elicitating plant resistance responses have been demonstrated and include surfactins and fengycins [49] and volatile organic compounds such as 2,3-butanediol [50]. Our preliminary results also indicate that *B. amyloliquefaciens* strain WG6-14 produces butanediol derivatives and that these volatile metabolites induce the expression of plant disease resistance genes such as those encoding phenylalanine ammonia lyase and pathogenesis related protein PR-1 in the leaves of rice plants (unpublished data). As another example, the control of *Cercospora* leaf spot on sugar beet by a phyllosphere-colonizing *B. mycoides* was attributed to its ability to induce systemic resistance [51].

In conclusion, our results demonstrate that *Bacillus* strains native to Taiwan, particularly strains WG6-14 and TKS1-1, can attenuate the symptoms and decrease the incidence of citrus bacterial canker disease. Because members of the *B. subtilis* group are GRAS bacteria, it would be safe to use these strains in the environment and maintain sustainability of the agricultural ecosystem. Biofilm formation as well as epiphytic colonization and survival are important for canker development in *X. axonopodis* pv. *citri*. The biocontrol efficacy of applying antagonistic bacilli to citrus leaves may be associated with their ability to interfere with colonization and biofilm formation by *X. axonopodis* pv. *citri*. Additionally, information obtained from molecular typing using the BOXA1R-PCR assay would provide DNA fingerprints valuable for patenting or commercializing these *Bacillus* strains.

## Materials and Methods

### Strains and growth conditions

The *Bacillus* and *Xanthomonas* strains and plasmids used in this study are listed in Table 1. *Bacillus* strains were routinely cultured on Difco™ potato dextrose agar (PDA, Becton Dickinson, Sparks, MD, USA) at 30°C. *Xanthomonas* strains were cultured on Difco™ Nutrient agar (NA; Becton Dickinson) or in trypticase soy broth (TSB; Becton Dickinson) at 27°C unless otherwise stated. When required, gentamicin (Gm; Sigma) was added to the medium at a concentration of 25 µg/ml. For the isolation of *Bacillus* strains, 1 gram of soil from the root rhizospheres of different plants, organic compost, potting mixes or natto was suspended in 5 ml of distilled water, heated at 80°C for 10 min, spread-plated on PDA and then incubated at 30°C for 1 day. *Bacillus*-like colonies were selected and tested for antagonistic activity against various phytopathogens including *Pythium aphanidermatum*, *Rhizoctonia solani* AG4, *Xanthomonas axonopodis* pv. *vesicatoria* XVT12, and *X. axonopodis* pv. *citri* XW19. Seven (HSP1, TKS1-1, OF3-16, SP4-17, WG6-14, TLB7-7 and WP8-12) of the 326 tested strains showed higher antagonistic activity than the remaining strains and were used for further study. To identify the *Bacillus* species, strains were subjected to physiological and biochemical characterization according to the methods described in Bergey's Manual [39]; they were identified using the Biolog system (Biolog Inc., CA, USA). For pathogenicity assays, *X. axonopodis* pv. *citri* strain TPH2 was generated by electroporating pBBR1MCS5 into *X. axonopodis* pv. *citri* strain XW19. For confocal laser scanning microscopy, pGTKan was electroporated into *X. axonopodis* pv. *citri* strain TPH2. Electroporation (12.5 kV/cm, 25 µF, 400 Ω) was performed using standard procedures [52].

### Antagonistic activity of *Bacillus* strains against *X. axonopodis* pv. *citri*

Antagonistic activity was determined using a dual culture assay. Twenty microliters of *X. axonopodis* pv. *citri* strain XW19 (optical density at 620 nm, OD_620_, of 0.3) grown in TSB at 27°C for 2 days and resuspended in sterile water was spread on an soybean yeast brown sugar agar plate (pH 7.5) (SYB agar containing 0.75% (w/v) soybean powder (Mayushan Foods Co., Ltd., Taiwan), 0.5% (w/v) yeast powder (Shin-Star Ltd., Taiwan), 2% (w/v) brown sugar (Cing-Liang Trading Co., Taiwan), 0.24% (w/v) K_2_HPO_4_ (Sigma), 0.03% (w/v) MgSO_4_·7H_2_O (Sigma), and 1.5% Bacto™ agar (Becton Dickinson), which was formulated to facilitate endospore formation). Twenty microliters of a *Bacillus* suspension (OD_620_ = 0.3) grown on an SYB agar plate at 30°C overnight and resuspended in sterile water was spotted inside a stainless steel ring (8 mm diameter) or on a paper disc (8 mm diameter) (Advantec, Tokyo Roshi Kaisha, Ltd., Japan) and placed on an SYB agar plate inoculated with *X. axonopodis* pv. *citri* strain XW19. The plates were incubated at 27°C, and the inhibition diameter of *Xanthomonas* growth was measured daily for 5 days.

### Sequence and phylogenetic analysis of *Bacillus* 16S rRNA

Genomic DNA was extracted from the *Bacillus* isolates using the Wizard® genomic DNA purification kit (Promega, Madison, WI, USA) according to the manufacturer's instructions. The 16S rRNA genes were amplified by PCR using the primers 8F and 907R according to the conditions described by Freitas *et al.* [24], except that 2× GoTag Master Mix (Promega) was used. The PCR products were then sequenced at the Automated DNA Sequencing Service Laboratory, National Chung Chung-Hsing University, Taiwan.

The 16S rRNA sequences were aligned using the Pileup program, SeqWeb version 3.1.2 (GCG Wisconsin Package, Accelrys Inc., San Diego, CA, USA). A distance matrix was generated by the Kimura 2-parameter method with the Dnadist program, Phylip version 3.6 (University of Washington, Seattle WA, USA). Phylogenetic trees were constructed using the neighbor-joining method (Neighbor program; Phylip version 3.6). The Seqboot program (Phylip version 3.6) was used to generate 1000 bootstrapped data sets. All sequences were compared with their respective type strains using the BLASTN program in the GenBank nucleotide database (http://www.ncbi.nlm.nih.gov/BLAST).

### DNA fingerprint and cluster analysis of *Bacillus* species

The genomic diversity of native and *Bacillus* type strains was assayed using molecular typing of the 16S–23S intergenic transcribed spacer region (ITS-PCR), tDNA-intergenic spacer polymorphism (tDNA-PCR) analysis, and repetitive element sequence-based PCR (rep-PCR) with the primers BOXA1R, ERIC, and (GTG)_5_ as described by Freitas *et al.* [24]. The primer sequences and amplification conditions were as previously described, except that 2× GoTag Master Mix (Promega) was used.

For cluster analysis, the similarity matrix was generated based on Jaccard's coefficient and was used to build a tree with the unweighted pair group arithmetic mean method (UPGMA) available as part of the UVP Vision Works LS 6.5 software (UVP, Cambridge, UK).

### Pathogenicity assay

*X. axonopodis* pv. *citri* strain TPH2 was cultured in TSB supplemented with 50 µg/ml gentamicin at 27°C, 100 rpm for 2 days; *B. subtilis* TKS1-1, and *B. amyloliquefaciens* WG6-14 were cultured in TSB at 27°C, 100 rpm for 1 day. The culture suspensions were adjusted to an OD_620_ of 0.3, and then sprayed on the leaves of Mexican lime in the greenhouse; the *Bacillus* suspensions were sprayed to the point of runoff 1 day prior to inoculation with *X. axonopodis* pv. *citri* strain TPH2. Milli-Q water was used as a control. The development of symptoms was recorded weekly for 1 month. The disease severity of citrus bacterial canker disease with and without *Bacillus* treatment was expressed as the number of cankers per cm^2^.

### Confocal laser scanning microscopy

*Bacillus* and *Xanthomonas* strains were cultured and inoculated onto leaves under conditions similar to those used for pathogenicity assays, except that *X. axonopodis* pv. *citri* strain TPH2 harboring pGTKan was used. Cells colonized on leaf surfaces were stained with acridine orange (0.025% in 0.026 M citric acid buffer [pH 6.6]; Sigma) and then examined with an Olympus Fluoview FV1000 confocal microscope (Olympus Optical Co. Ltd., Tokyo, Japan) equipped with an argon laser. Excitation and emission wavelengths were 510 nm and 488 nm, respectively.

### Efficacy of *Bacillus* endospore formulations on the reduction of the disease incidence of citrus bacterial canker

*Bacillus* endospore formulations were produced using established and patented methods [53] with a 750 liter liquid fermentor. Briefly, a single colony of *B. subtilis* TKS1-1 was inoculated into SYB broth and incubated at 30°C, 125 rpm overnight; this culture was used as seed inoculum for a large-scale preparation of endospores using liquid fermentation. Stepwise scaled-up fermentation was conducted in SYB in a series of fermentors at 30°C for 5 days with agitation at greater than 150 rpm and aeration rate greater than 1 air volume/culture volume/min. To assess its effect on the incidence of citrus bacterial canker disease, the endospore formulation was diluted 100-fold to achieve a final concentration of 10^9^ endospores/ml and applied 24 h pre- or post- inoculation with *X. axonopodis* pv. *citri* XW19. The leaves of navel orange trees were wounded with 20 pinpoint needle pricks per leaf. An *X. axonopodis* pv. *citri* XW19 suspension (OD_620_ of 0.3) was diluted 10-fold, then sprayed to the point of runoff on the wounded leaves in the greenhouse. Four treatments were conducted to evaluate the effect of the application time and frequency of *B. subtilis* strain TKS1-1 application on symptom development and disease incidence of citrus bacterial canker: (i) treatment T-B-T/1WK: the strain TKS1-1 endospore formulation was applied 24 h prior to *X. axonopodis* pv. *citri* XW19 inoculation, and then every week post-pathogen inoculation for 4 weeks; (ii) treatment T-B-D/1WK: the endospore formulation was applied 24 h prior to *X. axonopodis* pv. *citri* XW19 inoculation, followed by weekly spraying of Milli-Q water for 4 weeks; (iii) treatment D-B-T/1WK: the leaves were sprayed with Milli-Q water 24 h prior to strain XW19 inoculation, and then the strain TKS1-1 endospore formulation was spayed every week post-pathogen inoculation for 4 weeks; and (iv) treatment D-B-D/1WK: Milli-Q water was applied 24 h prior to XW19 inoculation and then every week post-inoculation for 4 weeks. The leaves treated with water were used as the control. Disease incidence (DI) at 4 weeks post-inoculation was calculated using the formula: DI(%) = (number of pinpoints with canker symptoms)/20×100.

### Statistical analysis

All experiments were performed at least three times. Data represent the means and standard deviations from at least four replicates of a representative experiment. The significant difference among treatments was analyzed by one-way ANOVA and Tukey's honestly significant difference (HSD) test using SPSS 15.0 software (SPSS Inc., Chicago, IL, USA).

### Nucleotide sequence accession numbers

The 16S rRNA sequences of the native *Bacillus* strains HSP-1, OF3-16, SP4-17, TLB7-7, TKS1-1, NT-2, WG6-14, and WP8-12 isolated in this study were deposited in the GenBank database (accession numbers HQ874610 to HQ874617).
